# BMC Clinical Pathology reviewer acknowledgement 2014

**DOI:** 10.1186/1472-6890-15-1

**Published:** 2015-01-28

**Authors:** Magdalena Morawska

**Affiliations:** BioMed Central, Floor 6, 236 Gray’s Inn Road, London, WC1X 8HB UK

## Abstract

The editors of *BMC Clinical Pathology* would like to thank all of our reviewers who have contributed to the journal in Volume 14 (2014).

**Jackeline Agorreta**

Spain

**Martin Almquist**

Sweden

**Federico Ambrogi**

Italy

**Gabriel Arismendi-Morillo**

Venezuela

**Sylvia Asa**

Canada

**Amanjit Bal**

India

**Maria G. Barderas**

Spain

**Alfredo Berruti**

Italy

**Fay Betsou**

Luxembourg

**Thomas Bleck**

USA

**Miroslav Blumenberg**

USA

**Ferdinando Carlo Maria Cananzi**

Italy

**Robert Canter**

USA

**Tai Chen**

USA

**Yoon-La Choi South**

Korea

**Maurizio Colecchia**

Italy

**Mark Coles**

UK

**John Cooper**

USA

**Giuliana Cortese**

Italy

**Dajun Deng**

China

**Mohammad Derakhshan**

UK

**Marie-Caroline Dieu-Nosjean**

France

**Jurek Dobrucki**

Poland

**Stefan Eichmuller**

Germany

**Andreas Erbersdobler**

Germany

**Gavino Faa**

Italy

**Aurelie Fabre**

Ireland

**William Funk**

USA

**Jiri Gallo**

Czech Republic

**Jerad Gardner**

USA

**Jeff Garner**

UK

**Francesco Gelsomino**

Italy

**Paul Goodfellow**

USA

**Tomasz Gosiewski**

Poland

**Jessica Grieger**

Australia

**Maria Guembe**

Spain

**Christopher Hall**

USA

**Kim Henriksen**

Denmark

**Rui Henrique**

Portugal

**Gerard Hornstra**

Netherlands

**Paul Hruz**

USA

**John Hughes**

UK

**Issei Imoto**

Japan

**Beatrice Gabriela Ioan**

Romania

**Rosalyn Irby**

USA

**Mitsuaki Ishida**

Japan

**Hiroaki Ito**

Japan

**Kiseok Jang**

South Korea

**Jacques Jani**

Belgium

**Peter Jensen**

Denmark

**Carmen Jeronimo**

Portugal

**Kamisha Johnson-Davis**

USA

**Fabio Klamt**

Brazil

**Mirjana Kocova**

Macedonia

**John Koethe**

USA

**Lingquan Kong**

China

**Rachel Koshy**

India

**Leda Kovatsi**

Greece

**Larry Kricka**

USA

**Cord Langner**

Austria

**Mariana Leal**

Brazil

**Jinming Li**

China

**Jose Antonio Lopez-Guerrero**

Spain

**Bertrand Ludes**

France

**Alexandre Manirakiza**

Central African Republic

**Federica Marchesi**

Italy

**Renato Mariani-Costantini**

Italy

**Vilma Martins**

Brazil

**Prashant Mathur**

India

**Fekicity Eb May**

UK

**Daniele Minardi**

Italy

**Hiroyuki Mineta**

Japan

**Glauco Miyahara**

Brazil

**Peter Molloy**

Australia

**Rodolfo Montironi**

Italy

**Sergio Morini**

Italy

**Mandi Murph**

USA

**Basavraj Nagoba**

India

**Luis Negrao**

Portugal

**Agnes Neuville**

France

**Alcina Nicol**

Brazil

**Krzysztof Okon**

Poland

**Ida Perrotta**

Italy

**Antoinette Perry**

Ireland

**Ruben Pio**

Spain

**Vito Pistoia**

Italy

**Peter Rambau**

Tanzania

**Khalil Razvi**

UK

**Christophe Redon**

USA

**Albert Reece**

Australia

**Andrea Renda**

Italy

**Richard Riedel**

USA

**Viviana Ritacco**

Argentina

**Ian Roberts**

UK

**Christoph Robier**

Austria

**Giulio Rossi**

Italy

**Felix Rückert**

Germany

**Ken Sakushima**

Japan

**Mario Scartozzi**

Italy

**Robert Schmidt**

USA

**Joel Schwartz**

USA

**Heidi Schwarzenbach**

Germany

**Nagamiah Selvakumar**

India

**Andrzej Semczuk**

Poland

**Shan-Rong Shi**

USA

**Hiroyuki Shimada**

USA

**Mohamed Shoukri**

Saudi Arabia

**Sveinung Sorbye**

Norway

**Hugo Sousa**

Portugal

**Marla Steinbeck**

USA

**Christopher Stevenson**

Australia

**Simona Stolnicu**

Romania

**Walter Storkus**

USA

**Ying-Hsiu Su**

USA

**Michael Tachezy**

Germany

**Masahiro Tachi**

Japan

**Hua Tang**

China

**Emina Torlakovic**

Canada

**Chang-Yong Tsao**

USA

**Toyonori Tsuzuki**

Japan

**Deniz Tural**

Turkey

**Jeroen F. Vermeulen**

Netherlands

**Semir Vranic**

Bosnia and Herzegovina

**Jim Wiley**

USA

**Sabine Windhorst**

Germany

**Kenneth Witwer**

USA

**Hua-Sheng Xiao**

China

**Ke Zen**

China

**Ming Hao Zheng**

Australia

**Daniele Zink**

Singapore

